# Elemental and micromorphological analysis of ion releasing restoration/carious dentin interface

**DOI:** 10.1038/s41598-025-13977-2

**Published:** 2025-08-21

**Authors:** Alaa Esmat Abdelsalam, Hoda Saleh Ismail, Hamdi Hosni Hamama

**Affiliations:** 1 Conservative Dentistry Department, Faculty of Dentistry, Mansoura University, Mansoura, Egypt; 2 Faculty of Dentistry, New-Mansoura University, New-Mansoura City, Egypt; 3 Faculty of Oral and Dental Medicine, Alsalam University, Tanta, Egypt

**Keywords:** Artificial caries-affected dentin, Dentin demineralization, Ion-releasing composite, Conventional glass ionomer, Elemental analysis, Micromorphological patterns, Restorative dentistry, Bonded restorations, Health care, Dentistry, Dental materials, Dental biomaterials

## Abstract

The objective of the current study was to evaluate elemental analysis and micromorphological patterns at the interfaces between tooth substrates either sound or demineralized dentin and ion-releasing restorations, including conventional glass ionomer and ion-releasing composite. The evaluation was conducted immediately (after 24 h) and after six months of artificial saliva storage. A total of 48 sound human premolars were used in this study. The premolars were randomly divided into two groups based on substrate type: sound and demineralized dentin (*n =* 24). Each substrate group was further subdivided into two subgroups according to restoration type: a conventional glass ionomer (Equia Forte HT) and an ion-releasing composite (ACTIVA-Bioactive Restorative) (*n =* 12). Each restoration subgroup was further divided according to aging condition: whether tested immediately after 24 h or after storage for six months in artificial saliva (*n =* 6). Specimens were sectioned buccolingually and longitudinally into two halves. For elemental analysis, one-half of each sectioned specimen was investigated using energy dispersive X-ray spectroscopy (EDX). Four elements (Ca, P, Al, and Si) were specifically examined at three different sites: dentin, hybrid layer, and restoration. For micromorphological patterns, two representative specimens from the second half of sectioned teeth were randomly selected to be observed under scanning electron microscope (SEM). EDX data were tabulated and subjected to statistical analysis, with a significance level set at *p* < 0.05. Kruskal-Wallis test revealed no statistically significant differences in distribution of the four elements across different tooth substrates or aging times. However, significant differences were observed based on the site factor. SEM micrographs revealed an ion-exchange zone in aged Equia Forte specimens and crystal-like structure deposition in artificially carious dentin bonded to ACTIVA. It has been concluded that both conventional glass ionomer restorative material and ion-releasing composite highlighted their bioactive behavior by demonstrating their potential ability to remineralize the demineralized dentin, reinforcing their promise as effective restorative options for dentin remineralization.

## Introduction

Concerns about minimally invasive Operative Dentistry have been raised regarding the preservation of caries-affected dentin (CAD) rather than its complete removal^1^. Although CAD is regarded as reactionary dentin, it is formed in response to caries stimuli and exhibits small alterations in cross-linking of its collagen fibrils^2^. It shows minimal demineralization, has remineralization capacity, and lacks bacterial contamination. Additionally, it possesses differences in morphological, chemical, and physical properties compared to sound dentin^3,4^. Although CAD is predominantly a clinical substrate for adhesive dentistry, reliable bonding to this substrate presents challenges due to low mineral content and altered organic matrix making adhesion more complex and resulting in lower bond strength than to sound dentin^5,6^.

Ion-releasing bioactive restorative materials are recommended for restoring CAD to increase bonding ability and facilitate the remineralization process^7^. These materials can promote mineral deposition and remineralization through releasing of calcium, phosphate, and fluoride ions, additionally, toxic effects of these ions can reduce biofilm penetration into marginal gaps^8^. Glass ionomers (GIs) are among the earliest bioactive restorative materials that can release and recharge fluoride ions^9^. A promising new generation of GI technology is (Equia Forte HT, GC Corporation, Tokyo, Japan), which is claimed by the manufacturer as a glass hybrid restorative material^10^. This product is reinforced by ultrafine highly reactive fluoroaluminosilicate glass fillers, along with a high molecular weight of the polyacrylic acid^11^. These fillers release more metallic ions, enhancing crosslinking of polyacrylic acid and resulting in a stronger matrix that improves physical properties^12^. It is a self-adhesive material that is chemically bonded to tooth structure thus, minimizing the microleakage. Additionally, it is characterized by high fracture toughness, flexural fatigue resistance, and flexural strength^13,14^.

Ion-releasing composite (ACTIVA-Bioactive Restorative, Pulpdent Corporation, Watertown, MA, USA) represents an innovative type of composite that combines attributes of composites, RMGIs, and GIs while minimizing their disadvantages^15^. The manufacturer claims that it contains a bioactive, shock-absorbing rubberized ionic resin (Embrace resin) matrix with a small amount of water and bioactive fillers that mimic chemical and physical properties of natural teeth. It does not contain bisphenol A, bisphenol A-glycidyl methacrylate (bis‐GMA), or BPA derivatives, which are responsible for polymerization shrinkage and stress^14,16^. It is characterized by its durability, wear and fracture resistance, chemical adhesion to teeth, and sealing against bacterial microleakage^14^. Additionally, it can respond to pH cycles by releasing and recharging high amounts of calcium, phosphate, and fluoride ions, resulting in formation of a phosphate apatite layer that seals the interface^14,17^.

A controversy has arisen regarding the self-adhesive property of this ion-releasing composite and whether ions released from restoration or tooth diffuse into the tooth/restoration interface when a resin adhesive layer is applied. Diffusion of ions is commonly observed between dentin and GIs, leading to formation of an ion-exchange zone that directly contributes to remineralization of carious dentin^18^. Hussein et al.^19^ demonstrated that the ion-releasing composite induced significant remineralization to demineralized dentin through mineral uptake, as confirmed by EDX data, without using an adhesive layer. Therefore, further investigations are needed to validate movement of these mineralizing ions across an adhesive layer.

Scanning electron microscopy with energy dispersive X-ray spectroscopy (SEM/EDX) approach offers both qualitative and semi-quantitative analysis that enables visualization of morphology of mineral deposits on the material’s surfaces and investigation of chemical composition at the tooth/material interface^20,21^. The technology of EDX is based on generation of characteristic X-rays that reveal presence of elements in the specimens^22,23^. Each element is thought to have a distinct fingerprint based on energy of the generated X-ray photons^24^.

Despite the previous data, a significant research gap remains regarding comprehensive evaluation of elemental composition and micromorphology of the interface between these materials and either sound or demineralized dentin, especially when an adhesive layer is used with the ion-releasing composite. Therefore, this study was conducted to evaluate elemental analysis and micromorphological patterns of tooth/restoration interfaces when conventional glass ionomer or ion-releasing composite were bonded to either sound or demineralized dentin. The tested null hypotheses were as follows: (1) there was no statistically significant difference in elemental analysis (Ca, P, Al, and Si) between the two tested restorative materials, either in sound or demineralized dentin and (2) there was no statistically significant difference in the effect of storage time on the elemental analysis of the two tested restorative materials, either in sound or demineralized dentin.

## Materials and methods

### Materials

Two restorative materials were utilized in this study: a conventional glass ionomer (Equia Forte HT, GC Corporation, Tokyo, Japan), and an ion-releasing composite (ACTIVA-Bioactive Restorative, Pulpdent Corporation, Watertown, MA, USA). Both were used according to the manufacturer’s instructions. Full details of the materials are presented in Table 1.

**Table 1:** Materials used in this study.

Material	Type	Composition	Manufacturer	Lot number
Cavity Conditioner	Surface Conditioner	20% polyacrylic acid, 3% aluminum chloride hexahydrate, distilled water	GC Corporation (Tokyo, Japan)	1912051
Equia Forte HT (Shade A2)	Bulk fill conventional glass ionomer	95% fluoro aluminosilicate glass, 5% polyacrylic acid powder, reinforced with silicate particles, 25–<50% polyacrylic acid, 5–<10% polybasic carboxylic acid, 5–< 10% tartaric acid	GC Corporation	2306132
Equia Forte Coat	Low-viscosity nanofilled resin	40–50% methyl methacrylate, 10–15% colloidal silica, 30–40% urethane methacrylate, 0.09% camphorquinone, 1–5% phosphoric ester monomer	GC Corporation	2304111
Tetric N universal bond	Universal adhesive	Phosphoric acid acrylate, HEMA, Bis GMA, urethane dimethacrylates, Ethanol, filmforming agent, catalysts, stabilizers	Ivoclar Vivadent AG, Schaan, Liechtenstein	Z04PGN
ACTIVA-Bioactive Restorative (Shade A2)	Ion- releasing Composite	A blend of diurethane and methacrylates with modified polyacrylic acid (44.6%), reactive glass filler (21.8 wt%), inorganic filler (56 wt%), patented rubberized resin (Embrace), and water	Pulpdent, Corporation, Watertown, MA, USA	230831

### Methods

#### Sample size calculation

GPower software (https://www.psychologie.hhu.de/arbeitsgruppen/allgemeine-psychologie-und-arbeitspsychologie/gpower) (Ver. 3.1.9.7; GPower, Kiel, Germany) was utilized to calculate the necessary sample size. The calculation was dependent on a previous study of a similar study design^25^. The mean and standard deviation of calcium content at dentin interface immediately and after 3 months of saliva storage for the same tested ion-releasing composite and universal adhesive were used for the analysis (3.1 ± 0.2 and 3.5 ± 0.2, respectively). The estimated sample size in each subgroup was (*n* = 6).

#### Teeth selection

A total of forty-eight sound premolars were extracted at the Oral Surgery Department Clinic as part of orthodontic treatment plan. The study protocol was approved by the Ethics Committee of the Faculty of Dentistry with an approval number (A04011023CD). After extraction, the premolars were cleaned of periodontal fibers and soft tissues using an ultrasonic scaler, examined for cracks under a stereomicroscope (SZ TP, Olympus, Tokyo, Japan), and then disinfected in a 0.5% chloramine T solution at a temperature of 4 °C. They were then stored in distilled water, which was changed weekly, and used within six months post-extraction^26^. The protocol for collecting and storing the teeth complied with the international and institutional guidelines for infection control.

#### Study design and specimen Preparation

All premolars were vertically embedded in self-cure acrylic resin (Acrostone, Anglo-Egyptian Company, Cairo, Egypt) blocks up to 2 mm below the cementoenamel junction (CEJ) within a plastic cylinder. A slow-speed diamond saw (Isomet 1000; Buehler, Lake Bluff, IL, USA) was used to horizontally section each tooth at the mid-crown under water coolant, removing the occlusal enamel and exposing the coronal dentin. The prepared dentin surfaces were examined under ×40 magnification using a stereomicroscope to confirm complete enamel removal. All procedures were performed by a single operator.

**Fig. 1:**
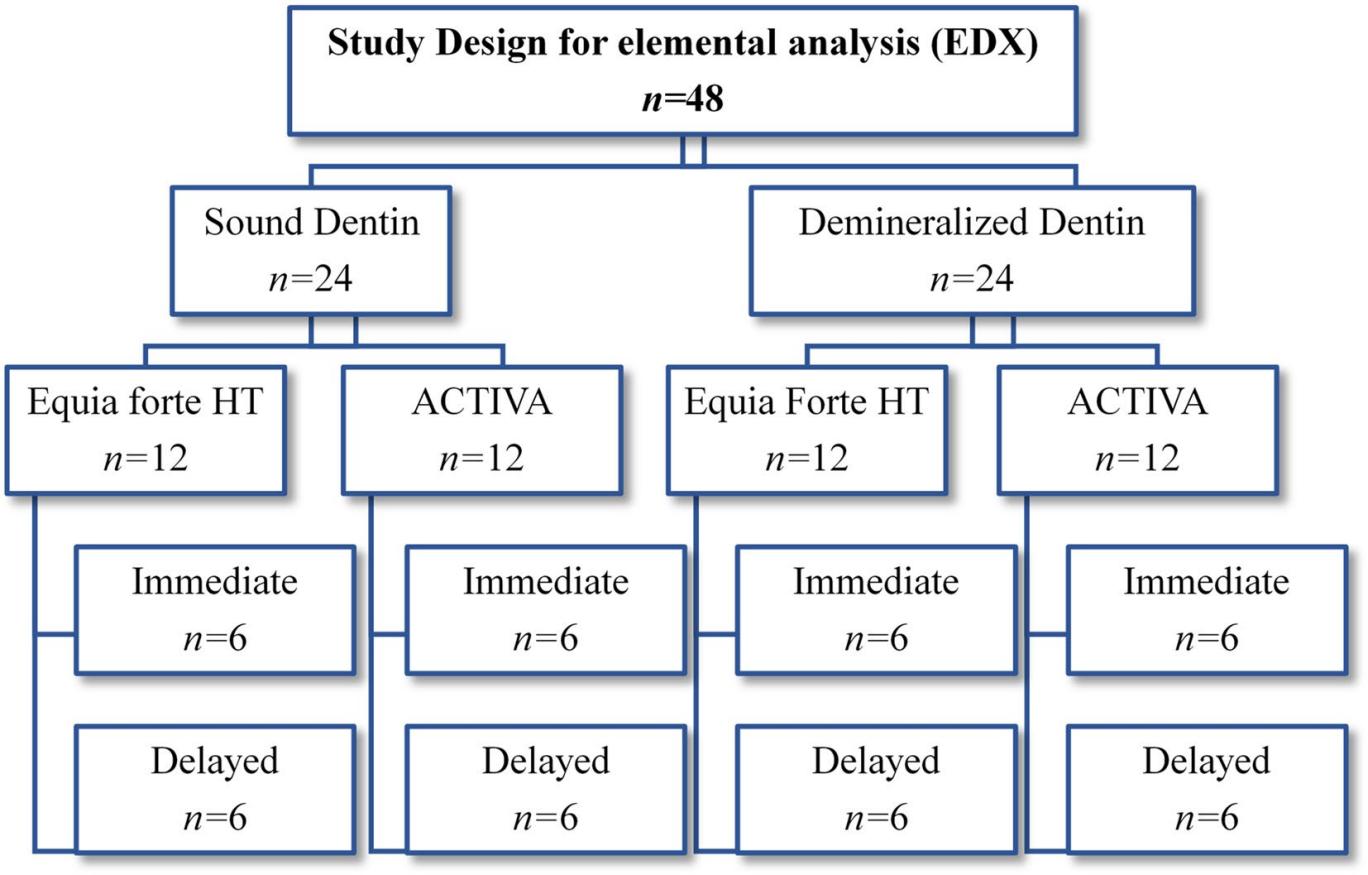
A schematic diagram illustrating the study design for elemental analysis of all studied groups.

Specimens were randomly divided into two groups based on dentin substrate. One group included sound dentin specimens (*n* = 24), while the other included demineralized dentin specimens (*n* = 24). Each substrate group was further subdivided randomly into two subgroups (*n* = 12) regarding the type of restorative material (Equia Forte HT and ACTIVA bioactive composite). A total of four subgroups were then randomly divided according to the aging condition (*n* = 6). Immediate specimens were tested after 24 h at 37 °C while the delayed ones were tested after storage in artificial saliva for 6 months at 37 ± 1 °C. The study design for elemental analysis of all studied groups is illustrated in Fig. 1.

#### Artificial demineralized dentin induction

For demineralized dentin specimens (*n* = 24), two coats of acid-resistant red nail varnish (Revlon, New York, NY, USA) were applied to all proximal walls, leaving the occlusal dentin surface exposed to demineralizing solution. Specimens were immersed in 40 mL of a demineralizing solution composed of: (1.5 mM of CaCl_2_, 0.9 mM of KH_2_PO_4_, 50 mM of acetic acid, and 0.02% of NaN_3_ with the pH value adjusted at 4.5 using NaOH) at 37 °C for 7 days^27^. The solution was replaced every 24 h to maintain a constant pH. The pH was periodically checked using a pH meter (STARTER 2100, OHAUS, Parsippany, NJ, USA). Following this, the nail varnish was removed and teeth were rinsed and placed in deionized distilled water (Direct-Q UV; Millipore, Molsheim, France) at room temperature until usage.

**Fig. 2:**
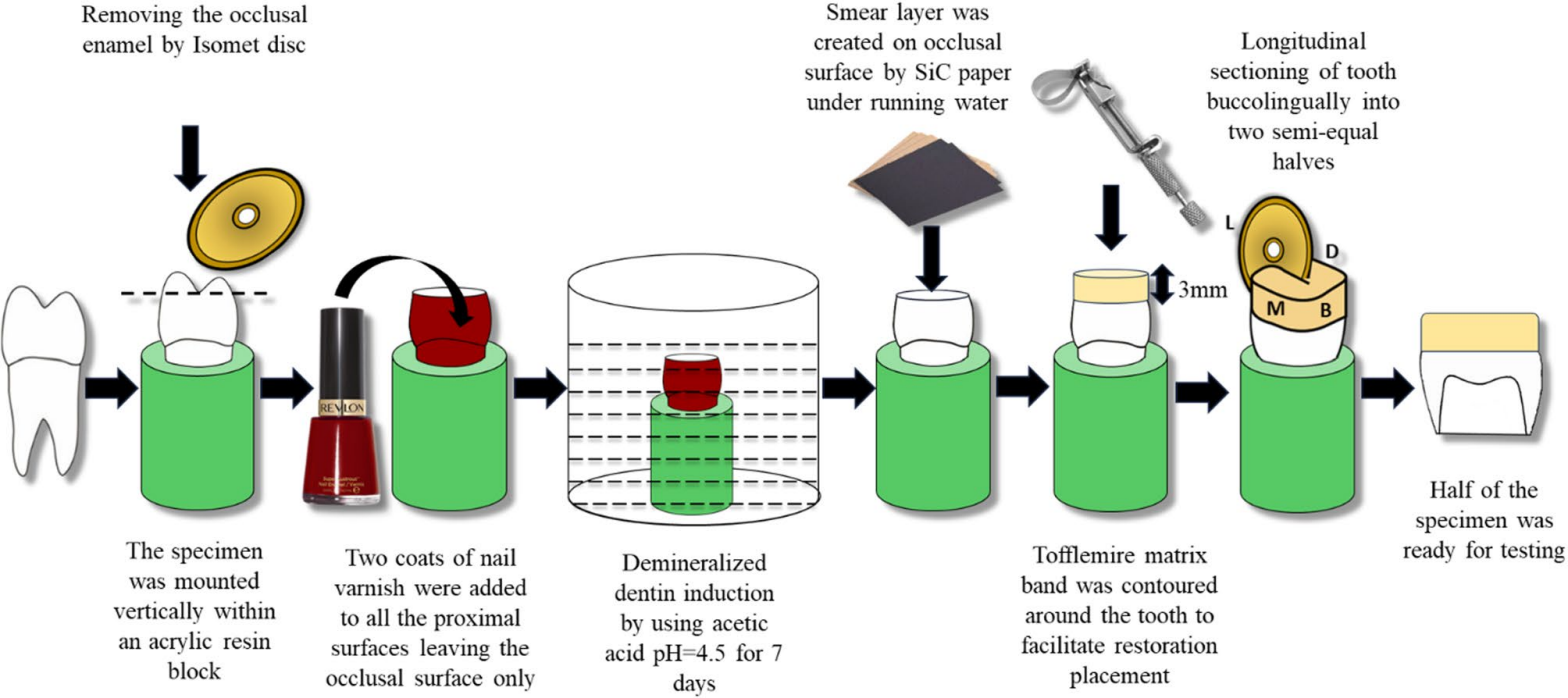
Schematic representation of the experimental procedures for induction of artificially demineralized dentin in the study.

All the exposed flat dentin surfaces, whether sound or demineralized, were polished manually using 600-grit silicon carbide paper (Fuji Star, Sankyo, Rikagaku, Saitama, Japan) under running water for 30 s to create a standardized smear layer^7^. All these procedures were performed by the same operator. A schematic representation of the experimental procedures for induction of artificially demineralized dentin is presented in Fig. 2.

#### Restorative procedures

A Tofflemire matrix band was contoured around each specimen to facilitate the restoration process. For Equia forte HT subgroups, a dentin conditioner (GC Corporation, Tokyo, Japan) was applied to dentin surfaces using a micro-brush for 10–15 s then rinsed and dried gently to avoid desiccation. The Equia Forte capsule was activated and mixed in an amalgamator (Softly 8, De Gotzen Amalgamator, Italy) for 10 s. Using a capsule applier (GC Corporation, Tokyo, Japan), the material was dispended directly on the occlusal dentin surface until it reached a thickness of 3 mm, then allowed to set. After removing the matrix band, Equia Forte Coat (GC, Tokyo, Japan) was applied to restoration surfaces and light cured for 20 s using LED curing light (COXO, DB-686 DELI, LED Curing light).

For ACTIVA subgroups, a universal adhesive (Tetric N Universal bond, Ivoclar Vivadent AG, Schaan, Liechtenstein) was applied in self-etch mode on dentin surfaces for 10 s, then it was air-thinned and light-cured for 20 s using LED curing light (COXO, DB-686 DELI, LED Curing light). The material was dispensed directly to the occlusal dentin surface from the syringe tip under slow steady pressure until reached 3 mm of material thickness, then it was allowed to be self-cured for 20–30 s and light-cured for 20 s using the previously mentioned light cure device. A radiometer (C10 curing light meter, Premium Plus UK Ltd, China) was utilized to check intensity of the LED curing light.

After the restorations were completed, half of the specimens in each restorative group were immersed in distilled water and stored in an incubator at 37 °C for 24 h before undergoing further testing. While the other half was subjected to artificial saliva storage in an incubator at 37 °C for 6 months. All restorative procedures were done by the same operator under magnification (×2.5 loupes, Amtech, Wenzhou, China) provided with LED headlight illumination and according to the manufacturer’s instructions.

#### Artificial saliva storage

Specimens in delayed groups were immersed in 40 ml of artificial saliva solution at 37 ± 1 °C using an incubator (BTC, Model: BT1020, Cairo, Egypt) for 6 months^28^. The artificial saliva formulation was based on McKnight-Hanes and Whitford but modified by exclusion of sorbitol^29^. The chemical composition of artificial saliva was: methyl-p-hydroxybenzoate (2 g/L), sodium carboxymethyl cellulose (10 g/L), MgCl_2_.6H_2_O (0.059 g/L), CaCl_2_.2H_2_O (0.166 g/L), KH_2_PO_4_ (0.326 g/L), K_2_HPO_4_ (0.804 g/L), and KCl (0.625 g/L) adjusted to pH = 7 with a weekly change to maintain the pH constant^30,31^.

#### Elemental analysis test

All specimens were sectioned buccolingually into two equal halves along the long axis using a low-speed diamond disc (Isomet 1000; Buehler, Lake Bluff, IL, USA) under water cooling. The sectioned surface of each bonded specimens was polished manually using 600, 1000, 1200, and 2000-grit SiC paper (MicrocutTM, Buehler, Lake Bluff, IL, USA) under water followed by using a polishing cloth with fine diamond pastes (6 μm, 4 μm, and 1 μm, respectively) (Diamat, Pace Technologies, Tuscon, AZ, USA)^32^. Debris was removed by using a digital ultrasonic cleaner (Guilin Woodpecker, Guangxi, China) for 5 min.

One-half of sectioned specimens either in immediate or delayed groups was subjected to elemental analysis by using EDX (Oxford software, X-Max 20, Abingdon, UK) under conditions of high vacuum field emission microscopy with a voltage of 20 kv, a working distance of 13∼15 mm, and a magnification power of ×1000. Four marker elements (calcium, phosphorous, aluminum, and silica) were detected at three different locations: dentin, hybrid layer, and restoration by using the ‘Area selection’ function to detect variations in mineral composition between intimately close areas^33,34^. The dentin areas were examined at a depth of 100–150 μm from the interface between the dentin and the restoration.

#### Micromorphological patterns at tooth/restoration interface

**Fig. 3:**
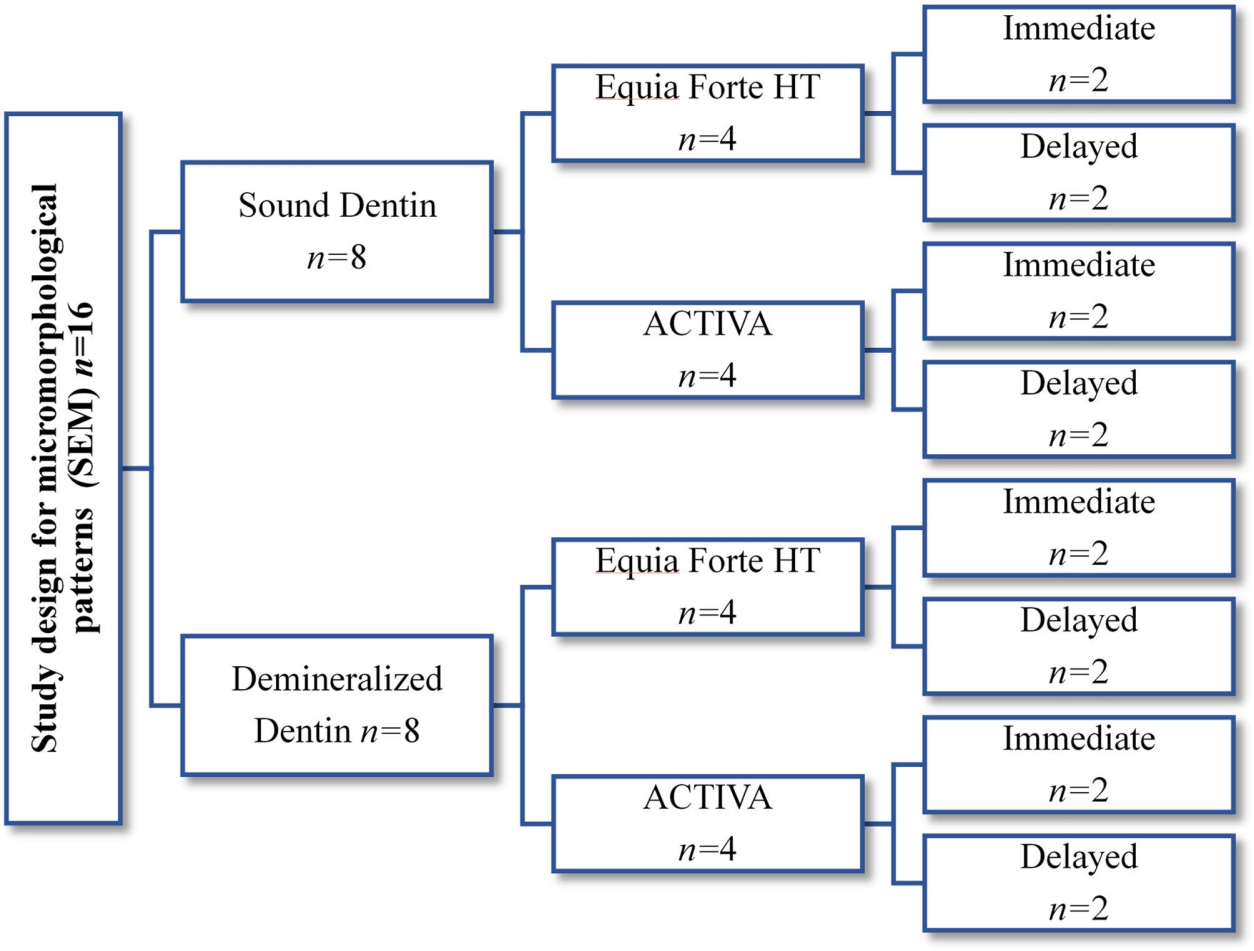
A schematic diagram illustrating the study design for micromorphological patterns of all groups.

Two representative specimens from each group’s second halves were analyzed using SEM (JSM-6510, LV, JEOL, Tokyo, Japan). After polishing and cleaning procedures, an acid-base challenge was performed by exposing the specimens to 10% orthophosphoric acid for 5 s, then rinsing with distilled water for 10 s, followed by immersion in a sodium hypochlorite solution (5%) for 5 min^7,35^. A gold layer was applied using a gold sputtering technique (SPI-MODULE ^TM^ Carbon / Gold Sputter Coater, EDEN instruments, Japan). The coated specimens were then observed in secondary electron detection mode with a voltage of 20–30 kV, a working distance of 9–13 mm, and a magnification power of ×2,000^36^. The study design for micromorphological patterns of all groups is presented in Fig. 3.

#### Statistical analysis

EDX data were statistically analyzed through using IBM SPSS (version 27, Armonk, USA). Shapiro Wilk test was used to check normal distribution of the data, which was found to be non-parametric. The data were represented by median and inter-quartier range. Kruskal-Wallis test was conducted to evaluate the effect of studied variables (tooth substrate, restoration type, aging, and site) on elemental analysis. The significance level was set at *P* ≤ 0.05. Mann-Whitney U test was used to compare distribution of significant element content (phosphorus and alumina) between two restoration types. A pairwise comparison test was conducted to identify which specific sites showed statistically significant differences in distribution of each element.

## Results

### Elemental analysis

Median and interquartile range values of Ca, P, Al, and Si levels for all studied groups are illustrated in Table 2.

Regarding the calcium ion content, Kruskal-Wallis test results showed no statistically significant difference in Ca ion distribution among different tooth substrates, restoration types, and aging times (p-values > 0.05). However, results revealed a statistically significant difference in Ca ion distribution across the site factor (p-value < 0.05). Pairwise comparisons of the site factor showed that Ca ion distribution was highest in dentin groups, followed by the hybrid layer, and lowest in restoration groups.

Regarding the phosphorous ion content, Kruskal-Wallis test results revealed no statistically significant difference in P ion distribution among different tooth substrates or aging times (p-values > 0.05). However, there was a statistically significant difference in P ion distribution between two restoration types and across different sites (p-values > 0.05). Mann-Whitney U test results revealed that the mean rank of phosphorus content was higher in ACTIVA restoration than Equia Forte. Pairwise comparisons of the site factor showed that P ion distribution was highest in dentin groups, followed by the hybrid layer, and lowest in restoration groups.

Regarding the alumina content, Kruskal-Wallis test results showed no statistically significant difference in alumina distribution among different tooth substrates or aging times (p-values > 0.05). However, a statistically significant difference in alumina distribution was observed between two restoration types and across different sites (p-values > 0.05). Mann-Whitney U test results showed that differences in mean ranks indicated that Equia Forte had a higher alumina content than ACTIVA restoration. While pairwise comparisons of the site factor revealed that alumina distribution was higher in restoration groups, followed by hybrid layer, and lowest in dentin groups.

Regarding the silica content, Kruskal-Wallis test results revealed no statistically significant difference in silica distribution among different tooth substrates, restoration types, or aging times (p-values > 0.05). However, a statistically significant difference in silica distribution was observed across different sites (p-values > 0.05). Pairwise comparisons of the site factor revealed that silica distribution was highest in restoration groups, followed by the hybrid layer, and lowest in dentin groups.

**Table 2:** Median and interquartile range values of ca, P, al, and Si levels for all studied groups.

Tooth substrate	Restoration	Aging time	Calcium	phosphorus	Alumina	Silica
Sound Dentin	Equia Forte	Immediate	0.247 (0.543)	0.156 (0.309)	0.269 (0.343)	0.326 (0.517)
		Delayed	0.348 (0.522)	0.212 (0.261)	0.239 (0.329)	0.201 (0.453)
	ACTIVA	Immediate	0.444 (0.497)	0.353 (0.251)	0.060 (0.101)	0.142 (0.634)
		Delayed	0.604 (0.504)	0.328 (0.248)	0.004 (0.122)	0.036 (0.600)
Demineralized Dentin	Equia Forte	Immediate	0.317 (0.564)	0.265 (0.327)	0.249 (0.364)	0.165 (0.531)
		Delayed	0.345 (0.496)	0.237 (0.265)	0.250 (0.348)	0.163 (0.426)
	ACTIVA	Immediate	0.211 (0.516)	0.230 (0.234)	0.015 (0.123)	0.544 (0.617)
		Delayed	0.504 (0.537)	0.320 (0.256)	0.015 (0.094)	0.156 (0.698)

### Micromorphological patterns of the tooth/restoration interface

**Fig. 4:**
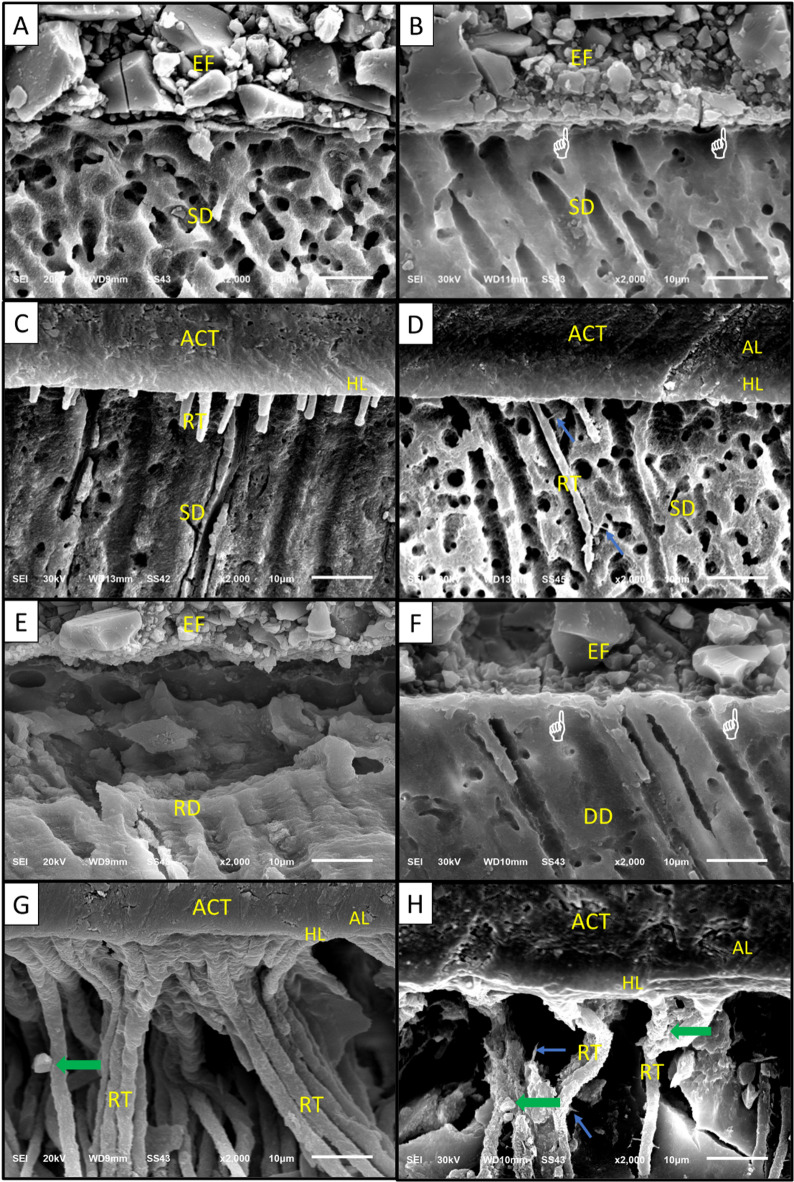
Descriptive SEM micrographs at the tooth/restoration interfaces of all studied groups. A: Sound dentin/Equia Forte/immediate group, B: Sound dentin/Equia Forte/delayed group, C: Sound dentin/ACTIVA/immediate group, D: Sound dentin/ACTIVA/delayed group, E: Demineralized dentin/Equia Forte/immediate group, F: Demineralized dentin/Equia Forte/delayed group, G: Demineralized dentin/ACTIVA/immediate group, H: Demineralized dentin/ACTIVA/delayed group. The finger pointer highlights a possible ion-exchange zone. The blue arrow indicates lateral branches protruding from the resin tags. The green arrow indicates possible mineral precipitates. EF: Equia Forte glass ionomer restoration, SD: Sound dentin, RD: remineralized dentin, ACT: ACTIVA bioactive restoration, AL: adhesive layer, HL: hybrid layer, RT: resin tags, and DD: Demineralized dentin.

Descriptive SEM micrographs obtained at the tooth/restoration interfaces of all studied groups at a magnification of ×2,000 to show surface morphology of different dentin specimens are presented in Fig. 4.

**Fig. 5:**
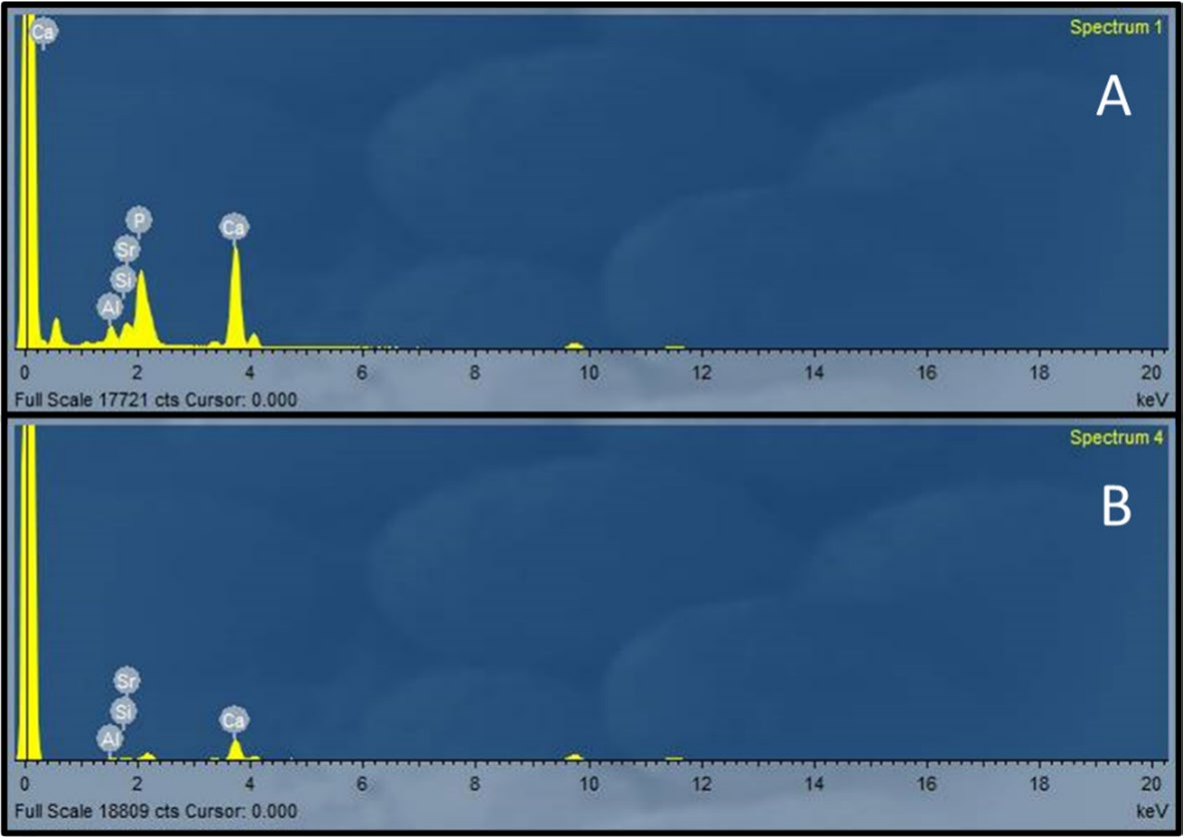
EDX spectra of a possible ion-exchange layer in the sound dentin/Equia Forte/delayed group and the demineralized dentin/Equia Forte/delayed group.

Regarding the sound dentin/Equia Forte/delayed group and demineralized dentin/Equia Forte/delayed group, SEM micrographs revealed a possible ion-exchange zone formed due to chemical adhesion between Equia Forte either with sound or demineralized dentin after aging. To confirm the presence of this zone, additional EDX analysis was performed on this area. EDX analysis confirmed presence of elements such as strontium, calcium, phosphorus, alumina, and silica at the tooth/restoration interface. These ions were released from GIs and tooth structure, leading to formation of what is known as the ion-exchange zone or interdiffusion zone. EDX spectra of a possible ion-exchange layer in the sound dentin/Equia Forte/delayed group and the demineralized dentin/Equia Forte/delayed group are shown in Fig. 5. Regarding SEM micrographs of demineralized dentin/ACTIVA/immediate and delayed groups, possible mineral precipitates were observed on the resin tags as shown in Fig. 4.

## Discussion

In the current study, the findings demonstrated that the elemental analysis, particularly (P and Al), was affected by the different restorative materials, either bonded to sound or demineralized dentin. However, there was no statistically significant difference in the distribution of Ca and Si in the two tested restorations, whether the dentin substrate. Consequently, the first null hypothesis was partially rejected. Independent of the other variable, outcomes of this study showed that the storage time had no effect on the elemental distribution of Ca, P, Al, and Si. Therefore, the second null hypothesis was fully accepted.

Ion-exchange is commonly observed when ion-releasing material is chemically bonded to tooth structure without using an adhesive layer. This has raised concerns in the literature that presence of a relatively impermeable resin layer may hinder ion diffusion and restrict the release of ions to the underlying tooth structure, particularly when a non-ion-releasing adhesive is used^25,36^. It has been suggested that permeability of the adhesive layer plays a major role in the ion-exchange process from ion-releasing restorations^25,36,37^. This raises the question of whether the use of an adhesive for certain ion-releasing materials affects diffusion of ions to the underlying tooth tissue. Therefore, this study sought to evaluate elemental analysis and micromorphological patterns between different restorative groups (conventional glass ionomer and ion-releasing composite) with different tooth substrates and aging conditions, especially when an adhesive agent was used for the ion-releasing composite.

Laboratory chemical models have been introduced for inducing artificially demineralized dentin, thus helping to evaluate the effectiveness of ion-releasing materials on demineralized dentin^38^. Artificially demineralized dentin was used as a critical comparable substrate to sound dentin due to a lack of standardization in natural caries-affected dentin and the great variability regarding the size, shape, and depth of natural lesions^39–42^. Based on the results of a recent systematic review^43^the second most commonly used protocol for induction of demineralized dentin was using of simple demineralization models including the use of acetic acid as a demineralizing agent. Therefore, this protocol was used in this study to create demineralized lesions that are relatively similar to natural ones^44^. The use of acetic acid is simple, cost-effective, and less time-consuming allowing reproducible experiments to be performed in a standard manner^44^. In the current study, the smear layer was created after the induction of artificial caries which is consistent with this Joves et al.^27^ and in contrast to others^6,39,45^. According to Prasansuttiporn’s study^46^CID reduction was performed using 600-grit SiC paper under running water to create a flattened smear layer covering the CAD surface, which may be clinically relevant.

Conventional glass ionomer was selected in this study as a control group for comparison, as its manufacturer claimed it is a glass hybrid restorative material, it can release mineralizing ions and has an antimicrobial effect^13^. Surface preconditioning with poly acrylic acid (PAA) is recommended by the manufacturer because this mild acid can partially dissolve the smear layer allowing phosphate and calcium ions from hydroxyapatite to remain attached to collagen fibrils, enabling GIC to adhere to tooth structures. This would improve the wettability of the adherent and increase the bond strength of the material to tooth structures^47,48^. Additionally, using a nanofilled resin-based coat, as suggested by the manufacturer, improves mechanical properties of the material and provides excellent sealing and marginal integrity, therefore, it enhances the material’s longevity and durability^49–51^.

Ion-releasing composite was selected in this study to evaluate its ion-releasing property since it is referred by the manufacturer as a bioactive material which has ability to release and recharge ions aiding in stimulating hydroxyapatite formation and promoting remineralization^52^. The use of ion-releasing composite with or without an adhesive agent was a controversial issue. In 2019, the manufacturer’s instructions recommended using an adhesive agent as a mandatory step because the tested ion-releasing composite doesn’t have self-adhesive property that was confirmed by Benetti et al.^53^. In addition to Rifai et al.^54^ who stated reported that using an ion-releasing composite directly without a bonding agent, resulted in the loss of restorations during fabrication of specimens^55^. Therefore, it was used in this study with an adhesive agent as suggested by the manufacturer, and in a self-etch mode as recommended by Sultan et al.^56^ and Tohidkhah et al.^16^. The low viscosity of self-etch adhesives allows partial demineralization of the smear layer that covers the dentin surface followed by infiltration of their acidic resin monomers into the dentin matrix resulting in formation of a hybrid layer^16^.

Elemental analysis was evaluated in this study using SEM/EDX which is considered as the most commonly used device for investigating elemental distribution and evaluating demineralization/ remineralization processes^24^. Each substrate or material has its own detectable marker, known as a “unique element”. Calcium and phosphate ions are unique markers for the tooth structure as they are necessary components for formation of hydroxyapatite layers on tooth surfaces^57^. Aluminum is an essential component in the glass ionomer material^58^. While silica is the unique element in the ion-releasing composite as it promotes remineralization of exposed dentinal tubules, enhancing crystallization of the material^34^.

Regarding the micromorphological analysis, SEM was utilized in the current study as it is one of the most frequently used devices for qualitatively assessing ultramicroscopic surface alterations on both enamel and dentin^59^. An acid/base challenge was performed to demineralize any dentin not infiltrated by resin, allowing dentin to be desiccated. Since orthophosphoric acid solution was used to demineralize dentin collagen fibers and sodium hypochlorite solution was applied to remove organic components, allowing for a clear visualization and characterization of the hybrid-like layer and enhancing features of the interface during imaging^60,61^. Gold coating is required for micromorphological analysis using SEM to prevent accumulation of static electric charge during electron irradiation, thereby improving the image resolution^62^.

A pilot study was conducted to confirm the effectiveness of the demineralizing protocol used. The results revealed that the calcium and phosphorus ion content in the demineralized dentin was found to decrease by 43.5% and 34.8%, respectively, compared to sound dentin within the first 50 μm into the dentin, reaching 32% for calcium and 29% for phosphorus at 150 μm when using the aforementioned demineralization protocol. According to Ngo et al.^63^ the lesion depth is determined when the mineral content in demineralized dentin reaches 95% of that in sound dentin. This finding validates the effectiveness of the demineralization protocol used in this study.

Elemental results revealed that distribution of Ca, P, Al, and Si within different tooth substrates was insignificantly different regardless of other factors. This can be attributed to ability of restorations to release Ca and P ions into the adjacent tooth structure, aiding in remineralization of demineralized dentin. Simeonov et al.^64^ and Raghip et al.^7^ reported that biomimetic remineralization can be achieved through deposition of minerals on the tooth surface via releasing of Ca and P ions when ion-releasing restorations are in direct contact with demineralized dentin^65^.

According to the restoration type variable, EDX results revealed an insignificant difference in Ca and Si distribution and a significant difference in P and Al distribution between two restoration types, regardless of other factors. For the ion-releasing composite, EDX results showed a significant increase in phosphate content compared to the glass ionomer material. This is due to its filler component and the unique bioactive ionic resin, which contains phosphate acidic groups that are responsible for continuous releasing and recharging of Ca and P ions^36,66^. Moreover, lacking of Bis-GMA in its chemical composition facilitates the ion release process which involves exchanging of sodium ions with hydrogen ions when the biomaterial is exposed to an aqueous environment^33,66^. This leads to releasing of calcium and phosphate ions from the material, allowing formation of mineral precipitates with ions from saliva thus, creating a calcium phosphate layer on the lesion surface^7,67^. Additionally, cleavage of Si-O-Si bonds in the silicate network results in bioglass dissolution, which causes a rapid increase in phosphate ion concentration^54,57^.

This finding is consistent with Kasraei et al.^57^ who observed that phosphate ion levels increased over time in the ion-releasing composite more than in GIs. This can be attributed to hydrolytic degradation of bioactive glass particles caused by presence of hydrophilic resins, such as HEMA or TEGDMA, in the ion-releasing composite. This degradation leads to an increase in phosphate ion concentration, which enhances apatite formation^57,68^. Similarly, O’Donnel et al.^69^ reported that apatite formation rate increased with a higher phosphate glass component. In contrast, Garoushi et al.^70^ found no evidence of mineralization potential in the ion-releasing composite, even though it released a relative amount of calcium.

Regarding the glass ionomer material, EDX results revealed a significant increase in alumina distribution compared to the ion-releasing composite. This can be attributed to different chemical compositions of the two materials. Alumina is an essential component of GIs, along with silica. Without alumina, GIs would lack reactivity and basicity, as silica alone cannot confer these properties^58,71^. Since the glass ionomer material releases ions at a slower rate due to increased matrix cross-linking, it promotes deeper remineralization of the lesion^49^. This aligns with Al-Wakeel et al.^34^ who concluded that the glass ionomer material is a promising restoration for demineralized dentin.

Regarding artificial saliva storage, results showed that storage time had no significant effect on elemental distribution of Ca, P, Al, and Si, regardless of other variables. This may be attributed to high viscosity of artificial saliva achieved by adding Methyl-p-hydroxybenzoate and sodium carboxymethyl cellulose in order to mimic mucin and protein content of natural saliva^72^ this high viscosity retards inward and outward movement of ions into the solution, as reported by Jingarwar et al.^73^.

Regarding the site factor, results showed that different sites significantly affected distribution of elements, regardless of all study variables. Ca and P content were highest in dentin groups, as these elements are fundamental to formation and deposition of hydroxyapatite crystals in the tooth structure and contribute significantly to the remineralization process^7,57^. Conversely, Al and Si content were highest at the restoration site, as these elements are key components in chemical composition of both the glass ionomer material and the ion-releasing composite.

According to SEM micrographs, a characteristic interfacial zone was observed in aged glass ionomer material specimens compared to the immediate ones. This ion-exchange zone was further validated through EDX analysis, which detected specific elements such as Ca, Sr, Al, Si, and P in this zone. Development of this layer depends on formation of hydrogen bonds between free carboxylic groups of PAA in the GI cement and the bound water on the tooth surface^71,74^. These hydrogen bonds are gradually replaced by stronger ionic bonds formed between cations, such as calcium in the tooth’s mineral phase, and anionic functional carboxylic groups in the cement. This process forms a chemically and mechanically strong acid-base resistant (ABR) layer at the GI/dentin interface^11,71,74,75^. The ABR layer serves as a protective barrier against demineralization by facilitating crystallization and ion deposition. Additionally, it is believed to enhance the long-term durability of the adhesive bond by reducing hydrolytic degradation and preventing leakage to the underlying tooth structure^33,48,74,76^. These findings align with those of Sarhan et al.^33^ and Ismail et al.^11^ who reported development of an ion-exchange layer in glass ionomer groups, with this layer being more prominent after extended storage time.

Since the glass ionomer material used is a strontium-based material by replacing calcium with strontium ions^12,77^. Presence of these elements at the interface is attributed to diffusion of calcium from the tooth mineral and strontium from the GI cement into the interfacial layer^74,76^. Since strontium and calcium have comparable chemical and physical properties, strontium can theoretically replace calcium in hydroxyapatite, thereby contributing to dentin remineralization^70,77^. Additionally, strontium enhances radiopacity of the glass matrix compared to calcium^71^.

SEM micrographs revealed formation of a hybrid-like layer of the ion-releasing composite. It was formed through penetration of resin monomers into exposed collagen fibers after partial demineralization of the smear layer by effect of the self-etch adhesive. This process preserves hydroxyapatite crystals to be attached around demineralized collagen, thereby producing micromechanical interlocking through formation of resin tags^4,34^. Additionally, SEM micrographs of the ion-releasing composite specimens bonded to demineralized dentin, compared to those bonded to sound dentin, showed precipitations of crystal-like structures on resin tags in demineralized specimens. This can be attributed to bioactive properties of the ion-releasing composite, which enable the material to stimulate apatite crystal formation at the interface^36,56,66^. These findings align with several studies^33,66,78^ that observed formation of an apatite crystal layer with the ion-releasing composite. In support of this, Ismail et al.^25^ stated that the semi-permeable adhesive layer allows diffusion of ions from the bioactive material into the tooth structure, extending into the interfacial zone.

Consequently, this laboratory study demonstrates that demineralized dentin can be remineralized by placing ion-releasing materials adjacent to artificial lesions. This is achieved through diffusion of released ions into both dentin and restoration through a semi-permeable adhesive layer in the case of the ion-releasing composite.

Some limitations of this study are represented by using a smaller sample size, which may reduce the representativeness of the results. Using SEM/EDX under a high vacuum led to some artifacts, such as microcracks and debonding at the tooth/restoration interface. The use of artificial demineralized dentin exhibits a limitation compared to natural lesions, however, this was attributed to a lack of standardization. Another recognized limitation in this study is not including such elements (Fl, Na, Cl, C, O, Mg) in the elemental analysis, which may provide more comprehensive and detailed information about the chemical interactions at the tooth/restoration interface. The authors recommend performing clinical trials to compare clinical performance of ACTIVA with other restorative materials on both enamel and dentin, particularly with ion-releasing adhesives.

## Conclusion

Based on the results, both conventional glass ionomer and ion-releasing composite facilitated formation of an ion-exchange layer and a hybrid-like layer with the underlying tooth structure, regardless of aging time. Additionally, both materials highlighted their bioactive behavior by demonstrating their potential ability to remineralize the demineralized dentin, reinforcing their promise as effective restorative options for dentin remineralization, particularly in minimally invasive dentistry. Future clinical trials should focus on validating the bioactivity of these restorative materials, assessing long-term outcomes, and evaluating their application in patients with demineralized dentin. These considerations will contribute to producing more clinically relevant research in restorative dentistry.

## Data Availability

The datasets generated and/or analyzed during the current study are not publicly available but are available from the corresponding author upon reasonable request.
